# Scientific production and thematic breakthroughs in smart learning environments: a bibliometric analysis

**DOI:** 10.1186/s40561-020-00145-4

**Published:** 2021-01-15

**Authors:** Friday Joseph Agbo, Solomon Sunday Oyelere, Jarkko Suhonen, Markku Tukiainen

**Affiliations:** 1 School of Computing, University of Eastern Finland, P.O. Box 111, FIN-80101 Joensuu, Finland; 2 Department of Computer Science, Electrical and Space Engineering, Luleå University of Technology, Luleå, Sweden

**Keywords:** Smart learning environments, Bibliometric analysis, Bibliometrix R-package, Science mapping, Research trends, Biblioshiny

## Abstract

This study examines the research landscape of smart learning environments by conducting a comprehensive bibliometric analysis of the field over the years. The study focused on the research trends, scholar’s productivity, and thematic focus of scientific publications in the field of smart learning environments. A total of 1081 data consisting of peer-reviewed articles were retrieved from the Scopus database. A bibliometric approach was applied to analyse the data for a comprehensive overview of the trend, thematic focus, and scientific production in the field of smart learning environments. The result from this bibliometric analysis indicates that the first paper on smart learning environments was published in 2002; implying the beginning of the field. Among other sources, “Computers & Education,” “Smart Learning Environments,” and “Computers in Human Behaviour” are the most relevant outlets publishing articles associated with smart learning environments. The work of Kinshuk et al., published in 2016, stands out as the most cited work among the analysed documents. The United States has the highest number of scientific productions and remained the most relevant country in the smart learning environment field. Besides, the results also showed names of prolific scholars and most relevant institutions in the field. Keywords such as “learning analytics,” “adaptive learning,” “personalized learning,” “blockchain,” and “deep learning” remain the trending keywords. Furthermore, thematic analysis shows that “digital storytelling” and its associated components such as “virtual reality,” “critical thinking,” and “serious games” are the emerging themes of the smart learning environments but need to be further developed to establish more ties with “smart learning”. The study provides useful contribution to the field by clearly presenting a comprehensive overview and research hotspots, thematic focus, and future direction of the field. These findings can guide scholars, especially the young ones in field of smart learning environments in defining their research focus and what aspect of smart leaning can be explored.

## Background

The evolution of learning and teaching methods from the traditional classroom learning environment to a technology-enhanced learning environment positively impacts education (Cárdenas-Robledo & Peña-Ayal, 2018; McIntosh, Herman, Sanford, McGraw, & Florence, 2004). This transition is even more relevant nowadays due to unforeseen circumstances that create an emergency on the world’s education, for example, where formal learning is not possible due to closure of schools as experienced in the recent COVID-19 pandemic (Atchison et al., 2020). As a result of this pandemic and to prevent the spread of the disease, many countries adopted online distance learning as an alternative teaching model (Reimers & Schleicher, 2020). This situation underscores the importance of developing a flexible, personalized, and adaptive learning environment to facilitate learning and teaching anytime, anywhere without physical contact and limited human interventions.

Research has shown that smart learning environments (SLE) can provide a twenty-first-century learning environment powered by advanced technology (Kim, Cho, & Lee, 2012; Laine & Joy, 2009), pedagogy (Tomczyk et al., 2019), and creative strategies (Harris, Dousay, Hall, Srinivasan, & Srinivasan, 2020). Thus, SLE promises to provide the future learning ecosystem by leveraging advanced learner models and evolving new technology. Smart learning environments refers to ubiquitous, context-aware, personalized, and intelligent system capable of providing a high level of motivation, engagement, and intelligent feedback for a better learning experience (Agbo et al., 2019; Hwang, 2014). The emerging field of smart learning environments began to gain scholars’ attention in recent times. The increasing growth of the field creates the opportunity to investigate the smart learning environments trends in the literature and how its discussion among scholars has progressed. A comprehensive review of literature in smart learning environments is very important. First, it will provide overview of the progress made by scholars and their status. Second, it will reveal critical information that can guide researchers in making decision regarding areas to focus their future research (field hotspots); and which publication outlet is suitable for publication. To this end, this study examines the research landscape of the smart learning environment to gain a comprehensive understanding of the research activities from a multidisciplinary perspective, trends, and possible future direction of the field.

Wang et al. (2020) recently conducted a related study that examined the research trend, status in the field of smart learning within China from 2012 to 2019. These authors, (Wang et al., 2020) were interested in knowing when research in smart learning began in China, its trend, and scholars’ publication contributions. The problem with this paper is that it is limited to a country and date bound. While our study derives motivation from Wang et al. (2020), it takes a different approach by conducting a comprehensive and all-encompassing study that is not limited to specific date ranges, regions, or countries. Besides, this study is focused on the science mapping of literature from the Scopus database by using the Bibliometric approach (Esfahani, Tavasoli, & Jabbarzadeh, 2019; Gilani, Salimi, Jouyandeh, Tavasoli, & Wong, 2019). Science mapping technic with Bibliometrix R-package is a useful approach to performing the Bibliometric analysis of scientific publications (Aria & Cuccurullo, 2017). A bibliometric study has been acclaimed to provide a useful tool for analysing the evolution of discipline based on its intellectual contributions, social, and conceptual structure (Zupic & Čater, 2015). Besides, many similar studies have applied bibliometric analysis to present an overview of specific field research. Among these studies, it is worthy of mentioning some recent and related areas such as research landscape of learning analytics (Waheed, Hassan, Aljohani, & Wasif, 2018), augmented reality research (Arici, Yildirim, Caliklar, & Yilmaz, 2019), multimedia learning research (Li, Antonenko, & Wang, 2019), and research on classroom dialogue (Song et al., 2019). These studies identified most outstanding publications, publication outlets, prolific scholars, research topics, and trends in the respective fields.

### Research objectives

This study aims to present a comprehensive review of the smart learning environment; hence, a bibliometric analysis is appropriate. To the best of our knowledge, no extensive bibliometric study of literature on smart learning environments has been conducted. This study is the first to conduct a bibliometric analysis of the field with a specific objective to examine the trend of smart learning environments over time; investigate the themes of smart learning in the publications; recognize prolific scholars and their contribution in the field of the smart learning environment; explore publication networks and collaborations across institutions, countries, and regions over time. Additionally, the study intends to identify any shift in the smart learning environment field’s boundaries from a large body of information in extant research.

The outcome of this study will provide useful knowledge for young scholars, mostly the young ones who are just starting to research in the field of smart learning environments. For example, young researchers can quickly identify top articles in terms of the number of citations, prolific authors, and research hotspots. Besides information such as trending topics and thematic future direction of smart learning environments can stimulate young researchers’ decision in terms of research interest. The main research question that this study seeks to answer is: *how research in the field of smart learning environments has progressed over the years in terms of scientific productions, thematic breakthroughs, scholars’ contributions, and future thematic direction?*

## Methods

In this study, a bibliometric mapping analysis was conducted. Bibliometric mapping is recently gaining more grounds in different disciplines (Aria & Cuccurullo, 2017; Arici et al., 2019; Song et al., 2019). Perhaps, the suitability of bibliometric for science mapping may have caused this extending acceptance among scholars (Aria & Cuccurullo, 2017). The entire procedure for conducting bibliometric mapping analysis in this study including data collection, screening, extraction, and synthesis are presented in this section.

### Literature search and data collection

First, we commenced by conducting a document search on the Scopus database. The search string consists of a combination of compound keywords concatenated with the OR operator. The first search field contained the keywords “smart learning environment” to search “All fields,” while the second search field contained keywords such as adaptive, context*, personalized, and intelligent. These additional keywords in the second line of the search field were selected because they are mostly used to define the characteristic features of smart learning environments (Hwang, 2014). Besides, these keywords have been associated with smart learning. For instance, Molina-Carmona and Villagr-Arnedo (2018) in their study entitled “smart learning”, emphasized keywords such as “personalized learning”, “adaptive learning, situation or context-aware learning as key orchestrates smart learning environments. The initial query without any filtering returned 1212 document results. The search and retrieval of the data were conducted on June 19, 2020. These results were later filtered to exclude some irrelevant items based on our inclusion and exclusion criteria. The inclusion and exclusion criteria are presented in Table 1. The search string combinations, operators, and filtering using the criteria explained in Table 1 is shown below.

**Table 1:** Inclusion and exclusion criteria for retrieving the dataset

	Code	Criteria	Comment
Inclusion criteria (IC)	IC 1	Articles containing one of the keywords in either title, abstract, or keywords.	This study conducted a search with five keywords concatenated with OR operator (see string combination above)
	IC 2	Documents written in the English language	Only articles written in the English language were considered in this study.
	IC 3	All date of publication	We did not specify date range since it is of interest to discover the trend of the field and when discussion among scholars began.
	IC 4	Articles in journals, conferences, and book chapters	The search is focused on documents published in journals, conferences, book chapters only
Exclusion criteria (EC)	EC 1	Articles with publication stage “in press.”	Only final articles that have been successfully published were considered in this study.
	EC 2	Articles whose source is a trade journal	This study considered articles from trade journals irrelevant since they do not go through the peer-review process. Trade journals are articles written majorly to educate, inform, or promote certain trade or industry. They are either published online or in newspapers and magazines.

(ALL(“smart learning environment”) OR TITLE-ABS-KEY(“adaptive context* personalized intelligent”)) AND (LIMIT-TO (LANGUAGE, “English”)) AND (LIMIT-TO(DOCTYPE, “ar”) OR LIMIT-TO(DOCTYPE, “cp”) OR LIMIT-TO(DOCTYPE,"ch”)) AND (EXCLUDE(PUBSTAGE, “aip”)) AND (EXCLUDE(SRCTYPE, “d”)) .

As the database was limited to Scopus, authors do not claim that an exhaustive list of data was acquired. The possibility of missing out on data from other databases such as Web of Science, PubMed, ERIC, etcetera could be minimal if a compatible formatting standard that allows for merging data generated from independent databases exists. Unfortunately, the bibliometrix R-package software^1^ used in this study does not currently support this ambition. However, Scopus covers a large number of articles and provides higher records in terms of citations (Heradio et al., 2016; Shen & Ho, 2020). Consequently, we claim that sufficient data to outline the scientific landscape, research hotspots, and other analysis conducted in this study was retrieved.

### Data extraction, loading, and conversion

In total, 1081 data were collected after refining based on the inclusion and exclusion criteria shown in Table 1. These data were exported for analysis. Thanks to Scopus platform for allowing an export up to 2000 data at a time, unlike Web of Science (WoS), where a maximum of 500 data can be exported per time. Besides, Scopus also allows scholars to export data to different file formats such as BibTeX, CSV, Plain Text, RIS formats, etc. In this study, data were exported in BibTex format, which is allowable for importing into biblioshiny for bibliometrix tools (Aria & Cuccurullo, 2017).

### Bibliometric analysis and software package

This study employed the use of bibliometrix R-package software, an open-source software that provides a set of tools for conducting quantitative research in bibliometrics. R-package was developed by Aria and Cuccurullo and written in the R language (Aria & Cuccurullo, 2017). It has the main algorithms for conducting statistical and science mapping analysis. The recent versions of bibliometrix R-package (i.e., 2.0 upwards) contains a web interface app (Biblioshiny) introduced to aid users without coding skills to conduct bibliometric analysis. Biblioshiny interface allows for data importing from Scopus or Web of Science databases in either BibTex, CSV, or Plain Text format. It is also possible to filter data in biblioshiny. Our study leveraged these opportunities inherent in biblioshiny for bibliometrix to import data from Scopus in BibTex format. The study analysis is presented in the result section.

### Data synthesis

Table 2 presents the summary information of the dataset. For example, the table revealed the numbers of document types in the data collected. Conference papers (*n*=497) are the highest number of the document type. Next is article papers (*n*=477), then book chapters (*n*=107). Other document types such as Notes, reviews, editorial, and short surveys accounted for the remaining 47.

**Table 2:** Data synthesis indicating primary information and summary of the dataset

Description	Results
Sources (journals, books, etc.)	535
Documents	1081
Average years from publication	2.48
Average citations per documents	4.46
Average citations per year per doc	0.99
References	38,382
Period	2002–2020
Keywords plus (ID)	3517
Author’s keywords (DE)	2885
Document Types	
Article	477
Book chapter	107
Conference paper	497
Authors	
Authors	2698
Author appearances	3578
Authors of single-authored documents	107
Authors of multi-authored documents	2591
Authors Collaboration	
Single-authored documents	130
Documents per author	0.40
Authors per document	2.5
Co-authors per documents	3.31
Collaboration Index	2.72

As used in this study, author’s keywords (DE) refer to a specific list of keywords authors of a publication have listed (usually less than ten) to describe what their study dwelt upon as used in the full-text. In contrast, keyword plus (ID) refer to extended keywords and phrases generated by Scopus system, which consist of keywords from the references cited by authors of a publication (Tripathi, Kumar, Sonker, & Babbar, 2018). In addition, authors per document refer to the mean number of authors per document, while co-author per document is the mean number of authors’ appearances per document—both authors per document and co-author per document measure authors’ collaboration.

## Results and discussions

Results and discussion of findings are presented in this section to reflect (i) growth and trends of smart learning environment research in terms of publication output, distribution, source, and citations; (ii) prolific scholars, affiliations, and social networks; (iii) thematic focus of the field of smart learning environments.

### Growth and trends of smart learning environments research

**Fig. 1:**
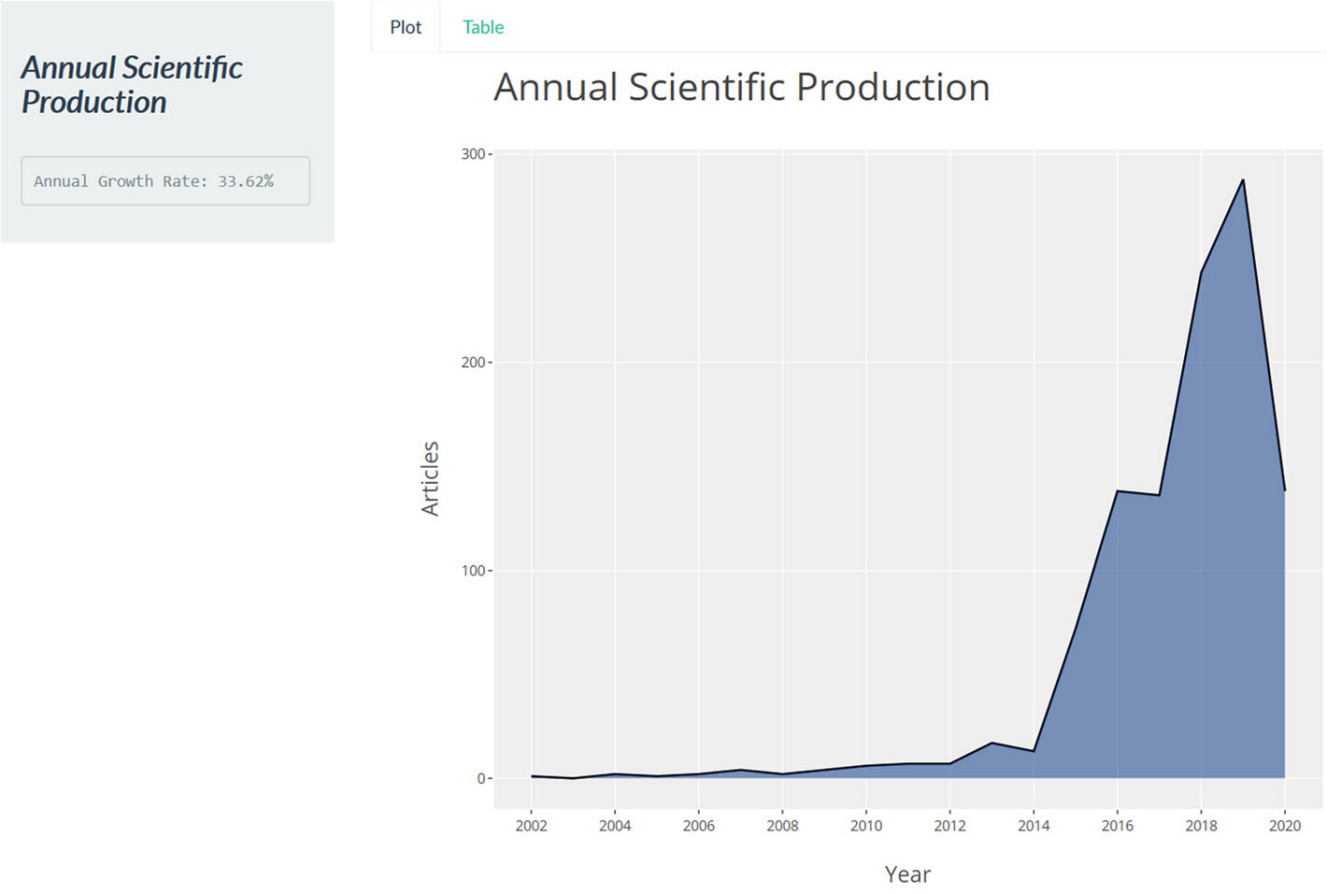
Annual scientific growth of smart learning environments: A compound annual growth rate computed by R- package, a geometric progression ratio with a constant scientific production rate over a period

In this section, we begin by presenting the annual scientific production of articles in the field of smart learning environments. As shown in Table 3, research in smart learning environments seems to commence in 2002 with the work of Sosteric and Hesemeier (2002) being the first and only article recorded in that year. Analysis from the bibliometrix R package shows that the field of smart learning environment has a 33.63% annual growth rate of scientific production from 2002 to mid-2020 (see Fig. 1). In 2015, 72 articles were recorded, which indicates the beginning of the impressive growth of publications in the field. This growth became drastic in 2016, where 138 articles were published. In 2019, 288 articles were published, which makes it the highest publication per year recorded so far. Since the field of smart learning environments is still emerging, it is expected, as revealed from the outcome of the analysis, that the scientific contribution would keep growing yearly.

**Table 3:** Articles production per year

Year	No. of scientific production
2002	1
2003	0
2004	2
2005	1
2006	2
2007	4
2008	2
2009	4
2010	6
2011	7
2012	7
2013	17
2014	13
2015	72
2016	138
2017	136
2018	243
2019	288
2020	138

Regarding the number of citations of smart learning environment publications, Table 4 presents the average citation per year. This result shows the amount of influence the publication has on the field per year. The result shows that the only single publication in 2002, which appears to be the beginning of the field, received an average number of 3.1 citations. This implies that the authors’— (Sosteric & Hesemeier, 2002)—work had a good impact in the field of smart learning environments. There was a dwindling of the number of citations between 2003 and 2009. However, the average citations per year grew to 10.2 in 2010, which is the highest citations recorded so far. Surprisingly, this number dropped sharply to 0.6 in 2011 and 0.7 in 2013. The reason for this fall in the citation in both years was not evident to authors; however, they can be considered as outliers. Besides, it can be seen from Table 3 that the annual scientific production in both years did not rise so much, which may have caused the decline in the annual citation for that year.

**Table 4:** Average citation per year

Year	Average citation
2002	3.1
2003	0.0
2004	0.2
2005	0.1
2006	1.5
2007	1.3
2008	0.8
2009	2.0
2010	10.2
2011	0.6
2012	6.8
2013	0.7
2014	1.0
2015	1.2
2016	1.7
2017	2.0
2018	1.7
2019	1.1
2020	0.0

### Relevant sources and documents of smart learning environment publications

**Fig. 2:**
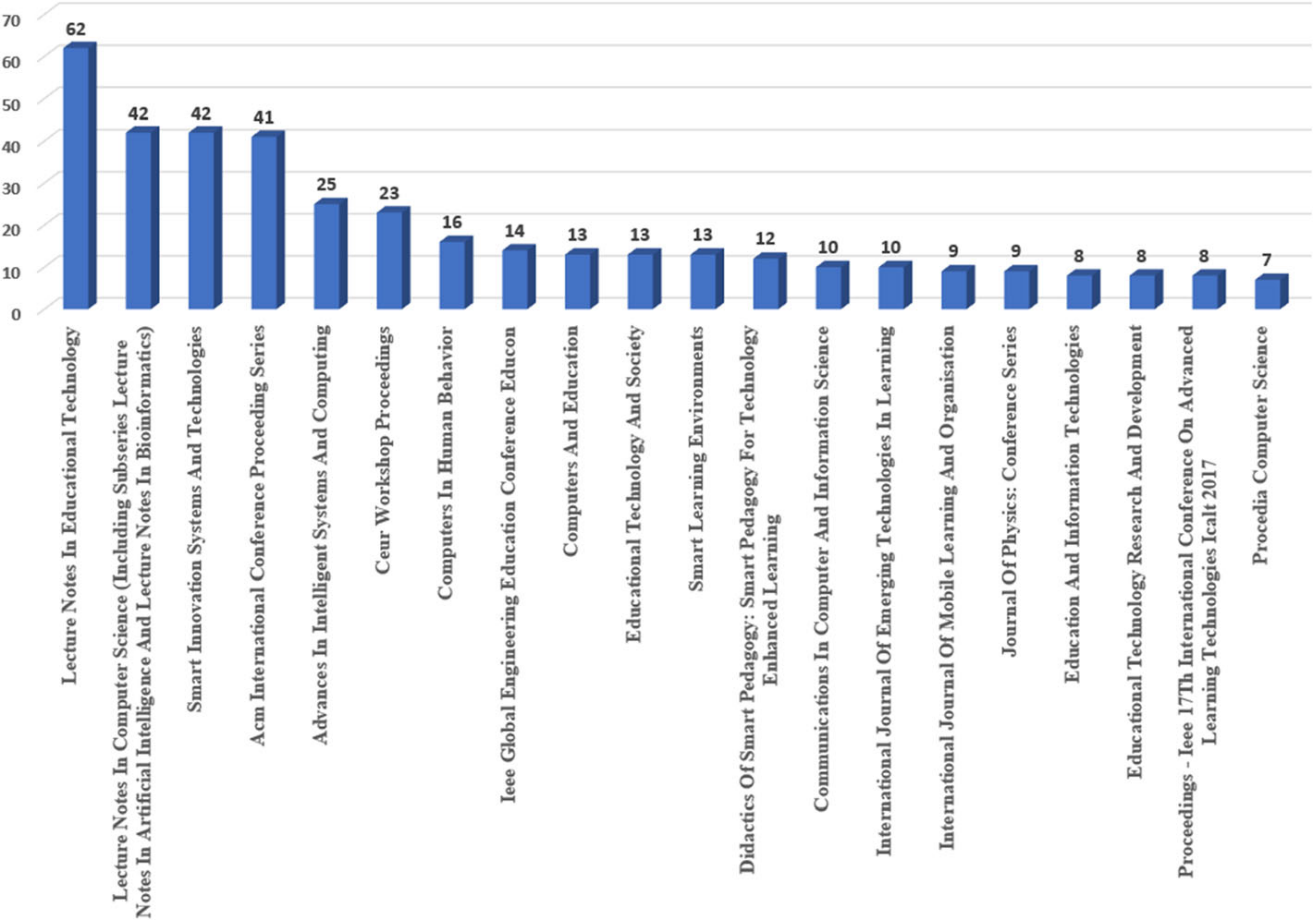
Distribution of articles by relevant sources from 2002 to mid-2020. (Based on this study’s Scopus dataset, lecture noted in educational technology remains the top source for smart learning environment publications)

In Fig. 2, the result of the top 20 most relevant sources focused on publishing articles on the smart learning environments is presented. This result is based on the data from Scopus retrieved in June 2020. It is shown that lecture notes on educational technology remain the topmost relevant source. Other relevant sources include Lecture Notes in Computer Science (Including Subseries Lecture Notes in Artificial Intelligence and that of Bioinformatics), Smart Innovation Systems and Technology, and Association for Computing Machinery (ACM) International Conference Proceeding series. Aside from these sources, dedicated journals shown by the analysis include Computers and Education, Educational Technology and Society, and Smart Learning Environments.

**Fig. 3:**
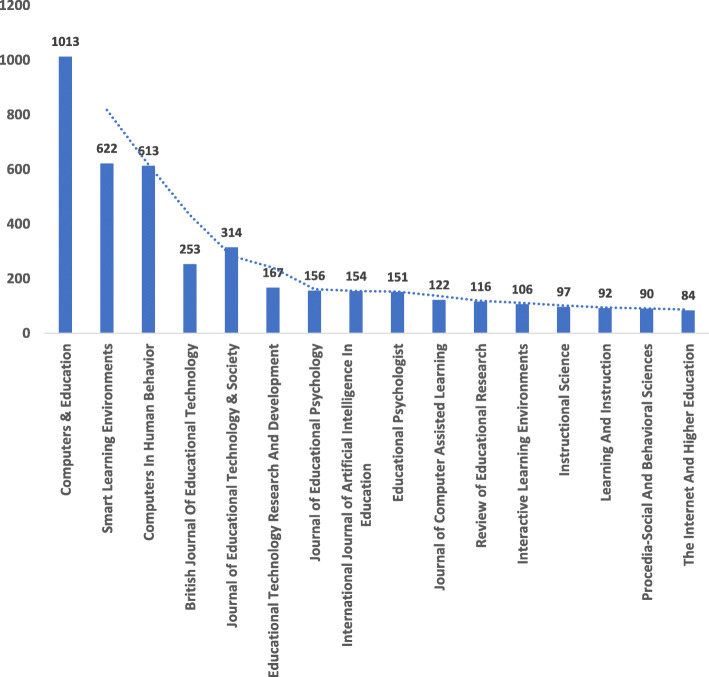
Relevant publishing outlets with most local citations: *Computers & Education*, *Smart Learning Environment*, and *Computers in Human Behaviour*s stands out

Among these top 20 relevant sources, further investigation (see Fig. 3) shows that “Computers and Education” is the most locally cited source with 1013 documents. Next, most locally cited source is the “Smart Learning Environment”—a fully open access journal initiated in 2014; published by Springer, and dedicated to providing opportunities for dialogue on the need for reform and innovative use of emerging technologies and pedagogy towards advancing learning and teaching in the twenty-first century (Spector, 2016). The smart learning environment has a total of 622 documents based on the dataset. Closely following in the list of most local cited resources is “Computers in Human Behaviour”, which has 613 documents.

Regarding the relevant document recorded in the field of smart learning environments, this study investigated the global and local citation of publications. Global citation measures the number of citations a document has received from the entire database, in this case, the Scopus database. The global citation also measures the impact of a document, which in most cases, could receive its larger number of citations from other disciplines. On the other hand, local citation measures the number of citations a document has received from documents included in the analysed data. The local citation also measures the impact of a document in the analysed collections (Aria & Cuccurullo, 2020). In other words, global citation considers citations from a global perspective in terms of disciplines, while local citation focuses only on citations within a discipline under study. Research has shown that aside from the scientific productivity counts, the number of citations for a publication also forms indices for ascertaining its significance and scholarly impact (Grant, Cottrell, Cluzeau, & Fawcett, 2000; Waheed et al., 2018). To this end, the analysis shows that the most globally cited paper between 2002 and mid-2020 came from the article published by Baker, D'Mello, Rodrigo, and Graesser (2010) with total global citations of 400. This authors’ work focused on the use of three different computer-based learning environments to teach students and, thereafter, investigated the incidence, persistence, and impact of their cognitive-affective states (Baker et al., 2010). In addition, the result shows 20 most cited documents from the study dataset (see Table 5). In the analysis, authors of Biblioshiny for Bibliometrix had written the algorithm to consider the local citation in order to determine the impact of documents within a dicipline. This study revealed that the work of Kinshuk, Chen N. S, Cheng I.L., and Chew S.W. published in 2016 top the list with local citation of 38 and global citation of 43. Suprisingly, Baker et al. (2010) that received massive global citations failed to show up among the top 20 most locally cited documents based on the dataset analysed. Out of the 1081 data collected in this study, Baker et al. (2010) was ranked 22 in the list of most cited documents with a total number of local citations of 4 and a total number of global citations of 400. The discrepancy in the number of local and global citations cannot be unconnected to the widely focused nature of these authors’ work—computerized learning environments—rather than the field of smart learning environments, which form a subset of their work.

**Table 5:** Top twenty most cited references based on number of local citations from the collection dataset

#	Document title	Authors & Year Published	Publication source	Local Total citation	Global Total citation
1	Evolution is Not Enough: Revolutionizing Current Learning Environments to Smart Learning Environments	(Kinshuk, Cheng, & Chew, 2016)	International Journal of Artificial Intelligence in Education	38	43
2	A Proposed Paradigm for Smart Learning Environment Based on Semantic Web	(Ouf, Abd Ellatif, Salama, & Helmy, 2017)	Computers in Human Behavior	32	37
3	Smart University Taxonomy: Features, Components, Systems	(Uskov et al., 2016)	Smart Innovation, Systems and Technologies	21	43
4	Three Dimensions of Smart Education	(Tikhomirov, Dneprovskaya, & Yankovskaya, 2015)	Smart Innovation, Systems and Technologies	11	29
5	Towards a Smart Learning Environment for Smart City Governance	(Hammad & Ludlow, 2016)	Proceedings - 9Th IEEE/ACM International Conference on Utility and Cloud Computing, UCC 2016	11	13
6	Identifying Potential Types of Guidance for Supporting Student Inquiry When Using Virtual and Remote Labs in Science: A Literature Review	(Zacharia et al., 2015)	Educational Technology Research and Development	9	44
7	Smart Learning	(Molina-Carmona & Villagr-Arnedo, 2018)	ACM International Conference Proceeding Series	9	3
8	Implementing Scenarios in a Smart Learning Environment	(Burghardt, Reisse, Heider, Giersich, & Kirste, 2008)	6Th Annual IEEE International Conference on Pervasive Computing and Communications, PERCOM 2008	8	11
9	Autotutor and Affective Autotutor: Learning by Talking with Cognitively and Emotionally Intelligent Computers that Talk Back	(D'mello & Graesser, 2013)	ACM Transactions on Interactive Intelligent Systems	5	145
10	Meta-Analysis of Inquiry-Based Learning: Effects of Guidance	(Lazonder & Harmsen, 2016)	Review of Educational Research	5	131
11	On the Way to Learning Style Models Integration: A Learner’s Characteristics Ontology	(Labib, Canós, & Penadés, 2017)	Computers in Human Behavior	5	23
12	Towards Competence-Based Learning Design Driven Remote and Virtual Labs Recommendations for Science Teachers	(Zervas, Sergis, Sampson, & Fyskilis, 2015)	Technology, Knowledge and Learning	5	9
13	Smart University: Literature Review and Creative Analysis	(Heinemann & Uskov, 2018)	Smart Innovation, Systems and Technologies	5	8
14	Developing A Smart Learning Environment in Universities Via Cyber-Physical Systems	(Lei, Wan, & Man, 2013)	Procedia Computer Science	5	8
15	Virtual Laboratories for Education in Science, Technology, and Engineering: A Review	(Potkonjak et al., 2016)	Computers and Education	4	187
16	Capturing Temporal and Sequential Patterns of Self-, Co-, and Socially Shared Regulation in the Context of Collaborative Learning	(Malmberg, Järvelä, & Järvenoja, 2017)	Contemporary Educational Psychology	4	37
17	Smart Pedagogy for Smart Universities	(Uskov, Bakken, Penumatsa, Heinemann, & Rachakonda, 2018)	Smart Innovation, Systems and Technologies	4	15
18	Supporting Adaptive Learning Pathways Through the Use of Learning Analytics: Developments, Challenges, and Future Opportunities	(Mavroudi, Giannakos, & Krogstie, 2018)	Interactive Learning Environments	4	11
19	ICT and Internet of Things for Creating Smart Learning Environment for Students at Education Institutes in India	(ur Rahman, Deep, & Rahman, 2016)	Proceedings of the 2016 6Th International Conference - Cloud System and Big Data Engineering, Confluence 2016	4	8
20	Innovative Maker Movement Platform For K-12 Education as a Smart Learning Environment	(Toivonen, Jormanainen, Montero, & Alessandrini, 2018)	Lecture Notes in Educational Technology	4	3

It is interesting to note also that a few studies in Table 5 received more local citations than global citations, as seen in the case of (Toivonen et al., 2018) and (Molina-Carmona & Villagr-Arnedo, 2018). One may think that the reverse should be the case. However, while authors cannot specifically give reasons for such a scenario, it might be the case of self-citations where these authors cited their study severally and published their works within the field of smart learning environments.

### Scientific publication production by region/countries

The study also conducted an analysis of scientific production (i.e., amount of publications) and contribution to the field of smart learning environments across regions/countries. The result demonstrates that the United States has the highest publication count from North America and closely followed by China from the Asia region. From Europe, the analysis shows that countries such as Spain, Germany, Greece, Finland, Italy, Netherlands, Turkey, and the Czech Republic contribute substantively to the field of smart learning environments. Australia is actively contributing to the field of smart learning environments from their region. However, in the of Africa region, the result shows that a few countries such as South Africa, Tunisia, Nigeria, Morocco, Ghana, and Tanzania are making some contributions to the field smart learning environments.

Further analysis shows the first 20 countries with total and average citations. The United States remains the top country, followed by China. However, surprisingly, Macedonia that seems invisible among the countries in terms of publication counts, became the third-ranked country in total citations and average citations of 188 and 62.7, respectively. This implies that although Macedonia may not have produced plenty of scientific articles in smart learning environments, the few published ones have a huge impact. Furthermore, as presented in Table 6, Germany and Finland are also among the top countries whose contributions in the field have a significant influence.

**Table 6:** Top twenty most cited countries in the field of smart learning environment

#	Country	Total Citations (TC)	Av. Article Citations
1	USA	998	39.9
2	China	203	6.8
3	Macedonia	188	62.7
4	Germany	128	10.7
5	Finland	115	8.9
6	Korea	91	2.3
7	United kingdom	61	8.7
8	Malaysia	54	13.5
9	Canada	48	16.0
10	Norway	43	7.2
11	Czech Republic	40	2.1
12	Belgium	29	9.7
13	Portugal	27	27.0
14	Romania	26	6.5
15	Greece	25	3.1
16	Spain	16	3.2
17	Ecuador	15	5.0
18	Italy	14	2.8
19	Netherlands	12	4.0
20	Turkey	12	12.0

### Prolific scholars, institutions, and collaboration network

#### Prolific scholars in the field of smart learning environments

**Fig. 4:**
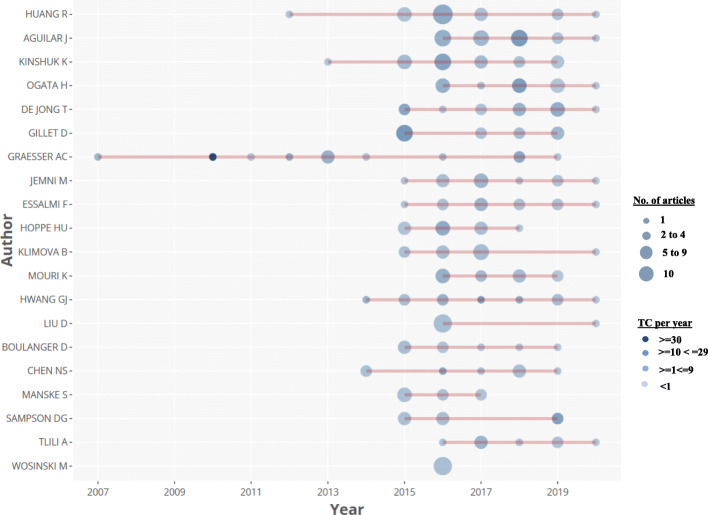
Top 20 authors productivity over the years: the line represents an authors timeline; bubbles size is proportional to the number of documents produced by an author per year; the color intensity of the bubble is proportional to the total number of citations per year; the first bubble on the line indicates when the author began to publish in the field; the bigger the bubble, the higher the number of articles published an author per year; bubbles with deeper color intensity indicates higher citation counts

Results from the top twenty most prolific scholars in the field of smart learning environments from 2002 to June 2020 based on the dataset are presented in Fig. 4. These scholars have shown consistency by contributing to the research body in this field. The result revealed that Arthur C. Graesser from the United States had produced a total of 12 documents and earned the highest citation counts of 618. He also has the highest h-index, which suggests that Graesser remains the most impactful author in the field of smart learning environments. Graesser’s first article was published in 2007 with total citations per year of 4.6. Although our result shows that Graesser has no publication yet in 2020, however, he has consistently published in this field between 2010 to 2014. The second most prolific scholar in this field is Jose Aguilar from Colombia. Aguilar has an h-index of 6 and a total of 20 publications. Aguilar began publishing in the field of smart learning environments in 2016, where he had six publications and consistently published 5, 6, 2, and 1 papers in 2017, 2018, 2019, and 2020 respectively. Similarly, the result shows that Menno D.T. de Jong from the Netherlands, Hiroaki Ogata from Japan, and Kinshuk from the United States have h-index of 6, 5, and 4 respectively based on our dataset; hence they have immensely impacted the field of smart learning environments. Other great scholars in this field and their scientific productions are shown in Fig. 4.

**Fig. 5:**
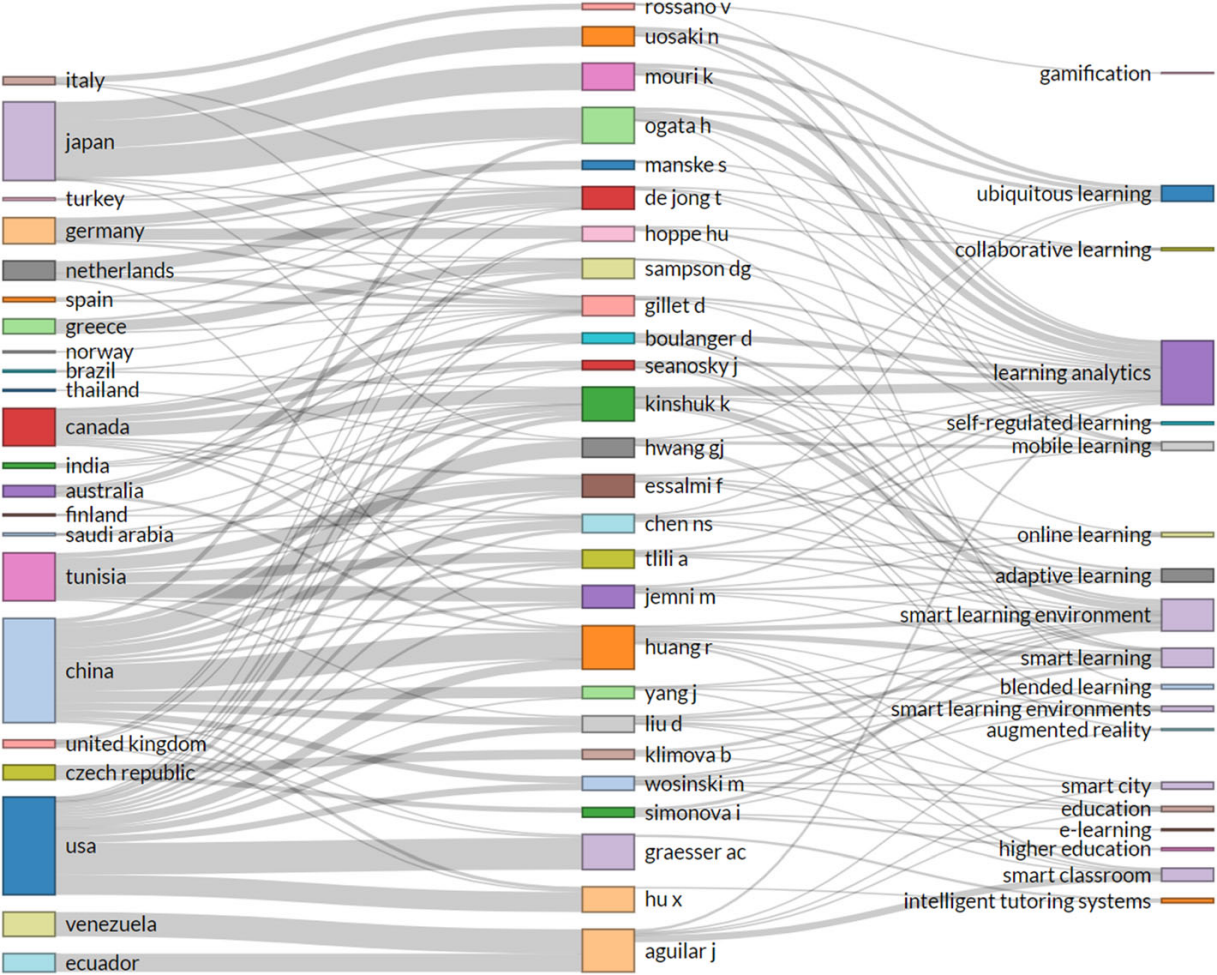
A three-field plot of countries, authors, and themes of smart learning environments: The emphasis is placed on the height of each box and thickness of the connecting lines; the taller the box, the more significant; and the thicker the lines’ correlation, the more information or volume of work was produced

A more visualized representation of prolific scholars vis-à-vis their countries and specific area of interest in the field of smart learning environments is shown in Fig. 5. This figure is a three-field plot of article contributions by countries, authors, and themes within the field of smart learning environments. The left-most column represents active countries, the middle column shows scholars’ names contributing from those countries, and the rightmost column represents the most used keywords by authors. The number of occurrences of these keywords forms what we refer to as ‘themes’ in this study. Note the height of the boxes and the thickness of the connecting lines. On the side of countries, China has more authors’ affiliations, with 120 authors connected to the country. Although our result revealed earlier that the United States is first in terms of scientific production and citation counts, they came second in authors’ affiliation. In that order, Japan has the next higher volume of authors, followed by Tunisia and Canada. Observing the thickness of the line leading from the countries to authors, we can see that Ronghuai Huang and Gwo-Jen Hwang remains the giant contributors from China. Similarly, Arthur C. Graesser and Xiaoqiang Hu are the main authors contributing to the field of smart learning environments from the United States. In Japan, Hiroaki Ogata, Kousuke Mouri, and Noriko Uosaki remain the prolific writers.

In addition, the aspect of learning analytic attracted more interest as the results show that 73 articles in learning analytics have emerged from authors such as Kinshuk, Hiroaki Ogata, and Kousuke Mouri, leading in that direction. Besides, the smart learning environment field also received interest and publications from Kinshuk and Ronghuai Huang as leading authors.

#### Institutions, co-authorship, and collaboration network

Regarding institutions and authors’ affiliations, contributing to the smart learning environment, the study investigated the publication output from the top 20 institutions. The result shows that Beijing Normal University, China tops with 37 documents. Next is the University of Memphis, in the United States, with a document count of 24. Athabasca University, Universidad De Los Andes, University of Hradec Kralove, the University of Twente, and Bradley University all belong to the top 20 institutions, with document numbers 22, 21, 20, 17, and 15, respectively (see Table 7).

**Table 7:** Most relevant institutions in the field of smart learning environment

#	Institutions	No. of Articles
1	Beijing Normal University	37
2	University of Memphis	24
3	Athabasca University	22
4	Universidad De Los Andes	21
5	University of Hradec Kralove	20
6	University of Twente	17
7	Bradley University	15
8	University of North Texas	14
9	University of Tunis	14
10	National Taiwan University of Science and Technology	13
11	Universidad Tcnica Particular De Loja	13
12	Arizona State University	12
13	University of Eastern Finland	12
14	University of Alicante	10
15	University of Duisburg-Essen	10
16	Kyoto University	9
17	National Sun Yat-Sen University	9
18	Osaka University	9
19	Universiti Sains Malaysia	9
20	Curtin University	8

**Fig. 6:**
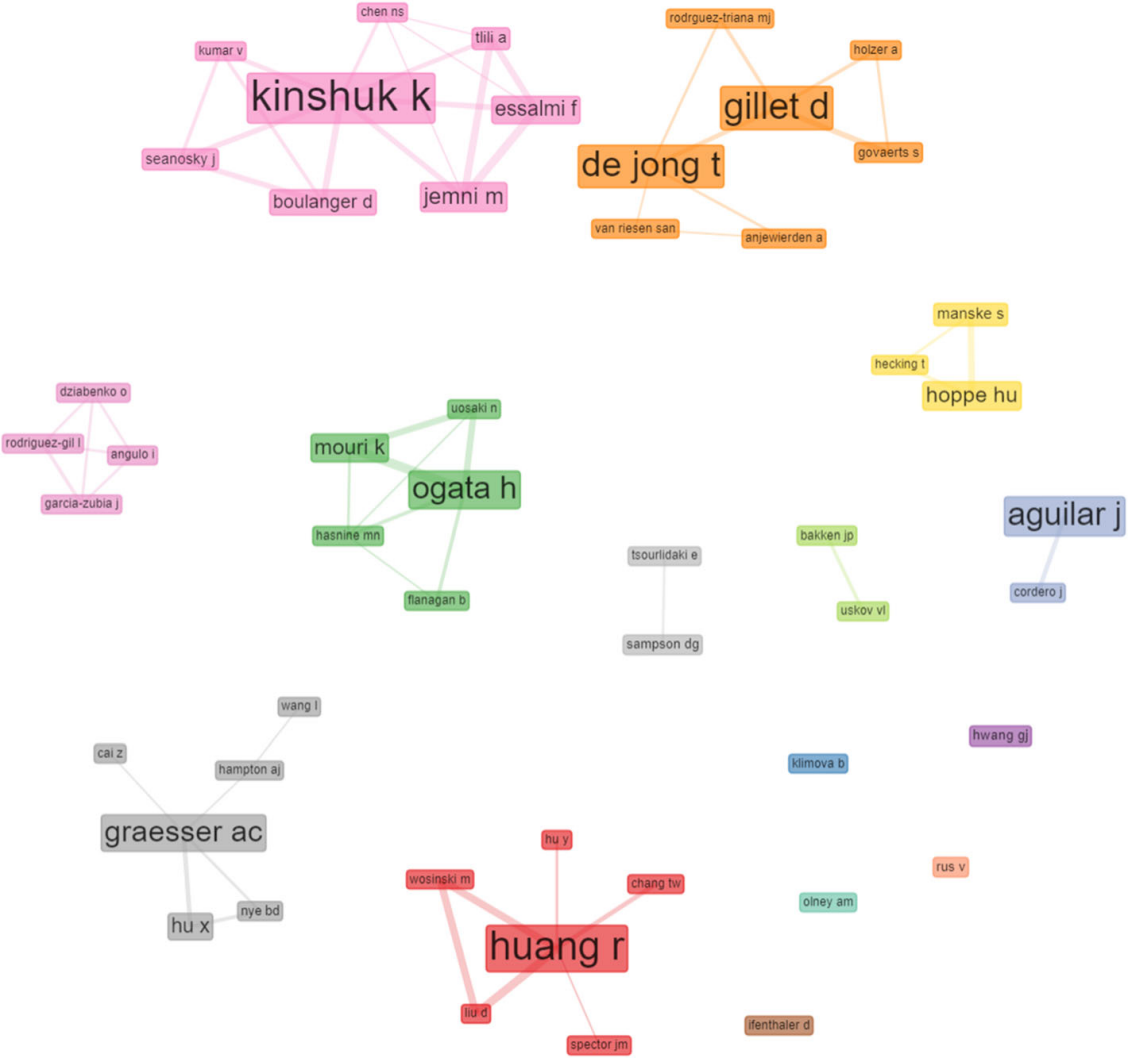
Mapping of authors’ collaboration network; Authors’ names are written in the boxes; the bigger the box, the wider the author’s collaboration network; also, there exist networks within a network, e.g., Fathi Essalmi and Mohamed Jemni all connected to Kinshuk

**Fig. 7:**
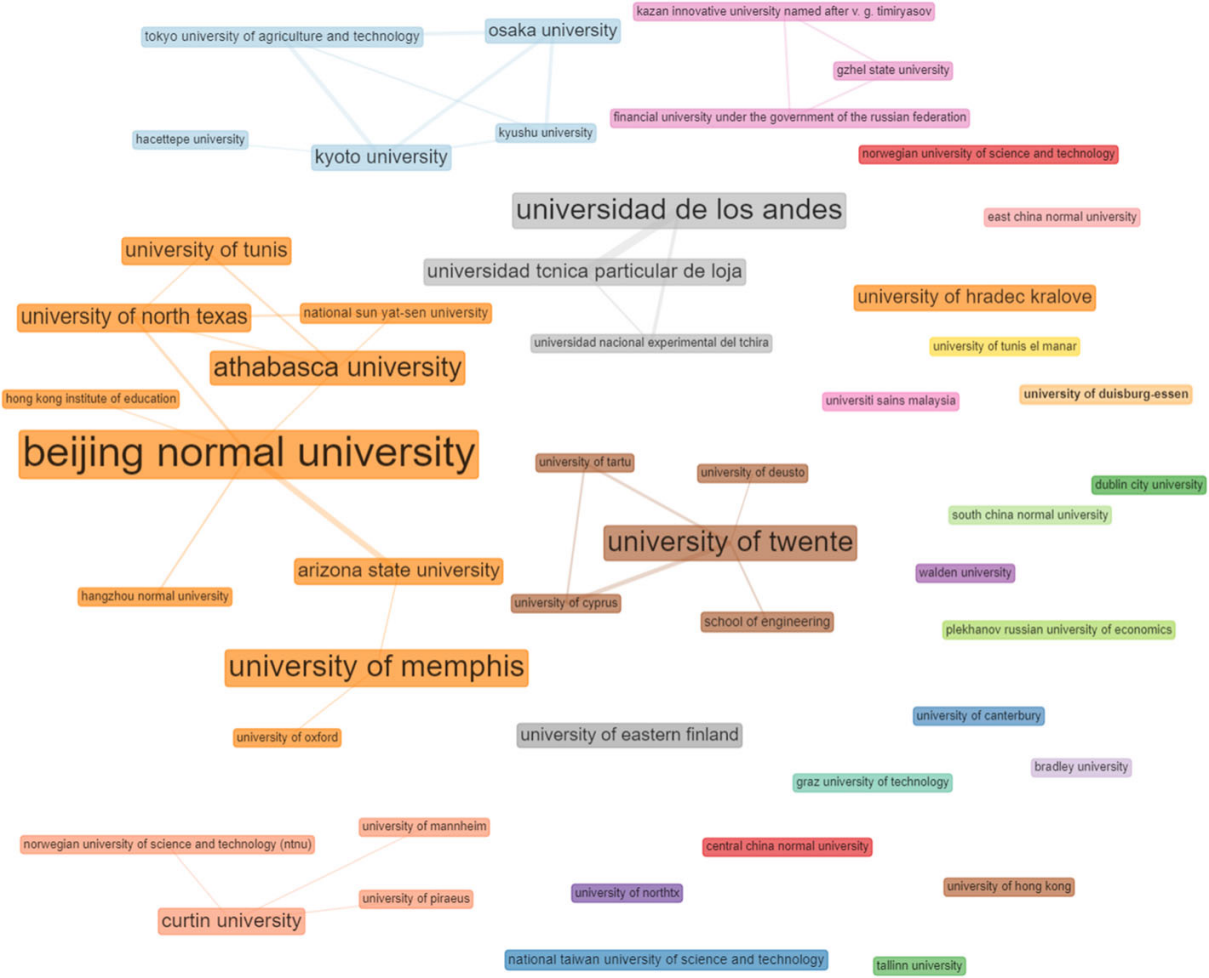
Mapping of institutions collaboration and social networks: clearly, institutions with a bigger network of collaborations are boldened while those with a few networks or none are smaller

Regarding co-authorship and social collaboration analysis, the study explored the social structure component of the bibliometrix R-package (Aria & Cuccurullo, 2017) provided in the biblioshiny user interface (UI). According to scholars, the social network of actors within a field delineates the relationship between two or more individuals, institutions, or countries with regards to collaborations (Prell, Hubacek, & Reed, 2009; Song et al., 2019). These relationships are presented in a network where nodes represent actors, and links connecting the nodes represent the relationships. In this study, we present the collaboration network between authors, as shown in Fig. 6, and the institution’s collaboration network, as shown in Fig. 7. The result shows that the big names already mentioned as prolific scholars in the field, such as Kinshuk, Huang, Graesser, Ogata, De Jong, and Aguilar are having a well-established collaboration network.

Similarly, institutions such as Beijing Normal University in China and the University of Twente in the Netherlands are seen to have created a big network of collaborations with other universities. For example, the Beijing Normal University has Arizona State University, Athabasca University, University of North Texas, Hong Kong Institute of Education, and Hangzhou Normal University in its network of collaborations. However, a few other universities are shown to have little or no collaboration network. Although these institutions are actively contributing to the research field of smart learning environments, they have not established collaborations with other institutions to expand their social network in the field. For example, Central China Normal University in China, the Graz University of Technology in Austria, the University of Eastern Finland in Finland, Bradley University in the United States, etcetera, are in isolation with no collaboration network.

### Thematic focus of the field of smart learning environments

This section investigates the themes that dominate the research landscape of smart learning environments and areas that scholars have focused on over the years. Besides, the study also tries to gain insight into whether there is a shift in the topic of discussion among scholars within the field. We first began by analysing authors’ keywords and their frequency of occurrences. Next, we carried out an analysis of keywords dynamics, trending topics, co-occurrence network, and thematic areas of the field.

#### Keywords analysis, co-occurrence network, and trend topics

**Fig. 8:**
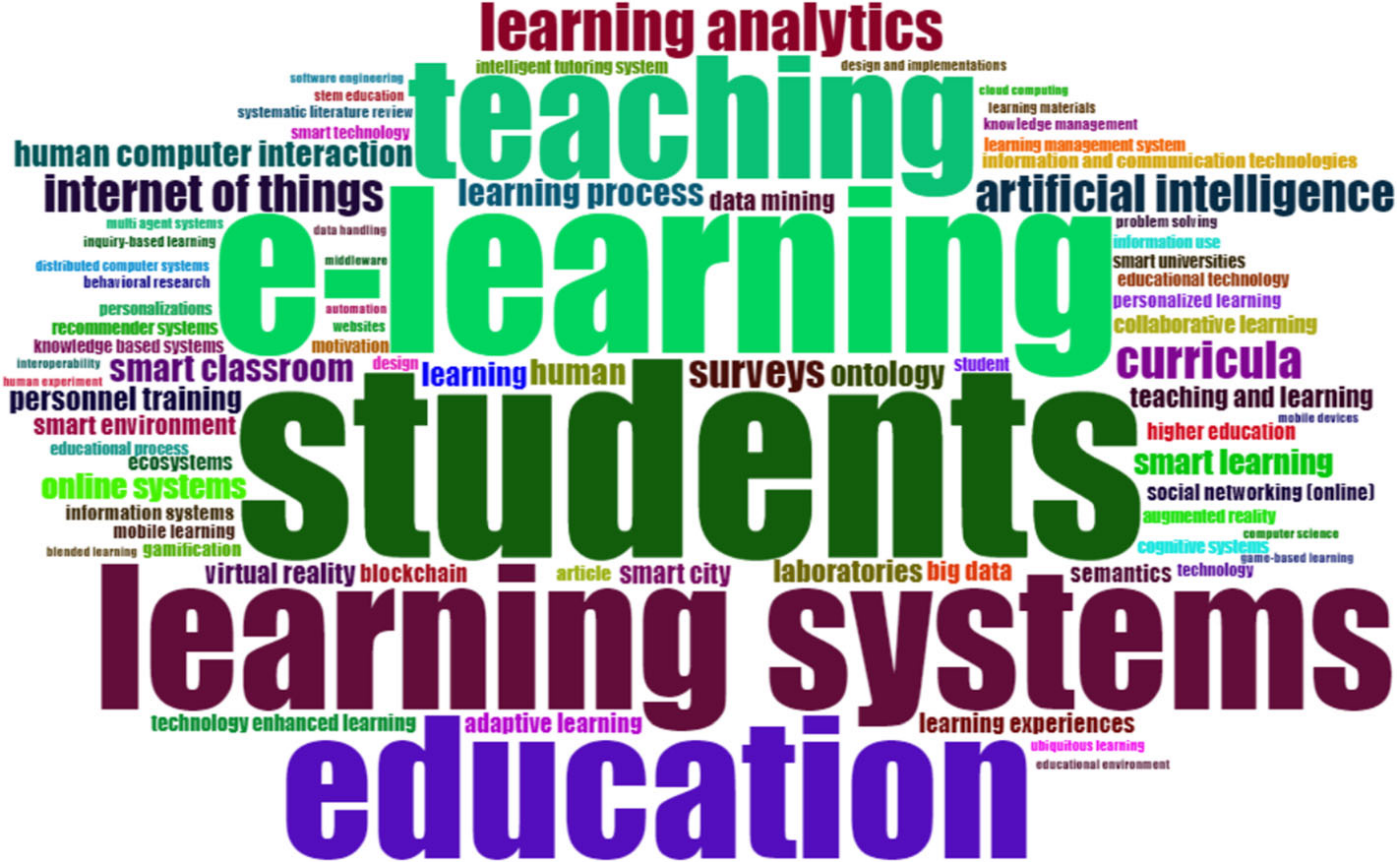
A Visualized Word-cloud of frequently used keywords in the smart learning environment field: these are among the highest number of repetitive keywords within the field

Analysis of keywords used by authors in publications is an essential tool for investigating trending topics and scholars focus in the field (Song et al., 2019). This analysis is so because publication keywords help to identify the topic and focus of that publication quickly. The word-cloud in Fig. 8 shows frequently used keywords in smart learning environments publications.

**Fig. 9:**
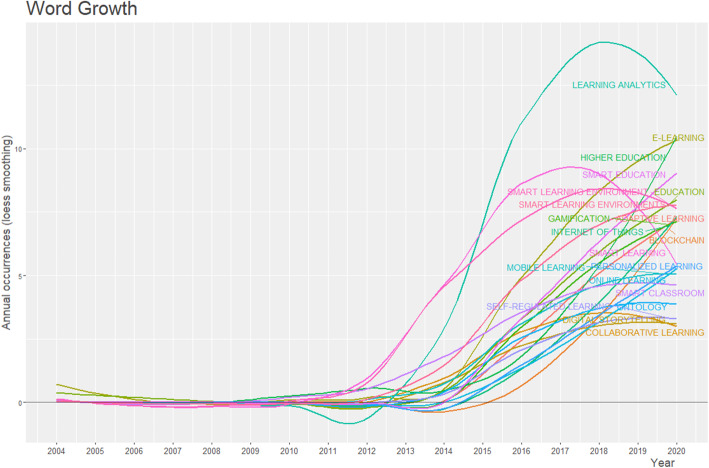
Authors’ keywords dynamic view over time: it shows the growth of keywords*; learning analytic* has grown till 2018 and began to decline thereafter; however, *higher education, personalized learning, internet of thing,* and *blockchain* are keywords that show upward growth as of 2020

Specifically, Fig. 9 is a visualized word dynamics of the authors’ most used keywords. As shown in the figure, most of these keywords began to appear in the research landscape around 2010 and continued to grow afterward. While a few of them, such as “*smart learning environment*” and “*smart learning environments*,” began to witness a rapid growth after 2011, *learning analytics* had a negative trend.

However*, learning analytics* became one of the most used keywords from 2013 and grew very fast until 2018. This finding signified that *learning analytics* became the most discussed topic as an aspect of smart learning environments among scholars within those years. Notably, between 2018 and mid-2020, when this study was conducted, the use of these keywords began to nosedive. However, frequently used keywords such as *higher education, online learning, smart education, adaptive learning, and personalized learning* existed from around 2004 but began to rise after 2010. Between 2010 and 2020, keywords such as *blockchain and internet of things* emerged and continued to grow (see Fig. 9). This finding suggests that the field of smart learning environment will continue to be researched around these prevailing aspects.

**Fig. 10:**
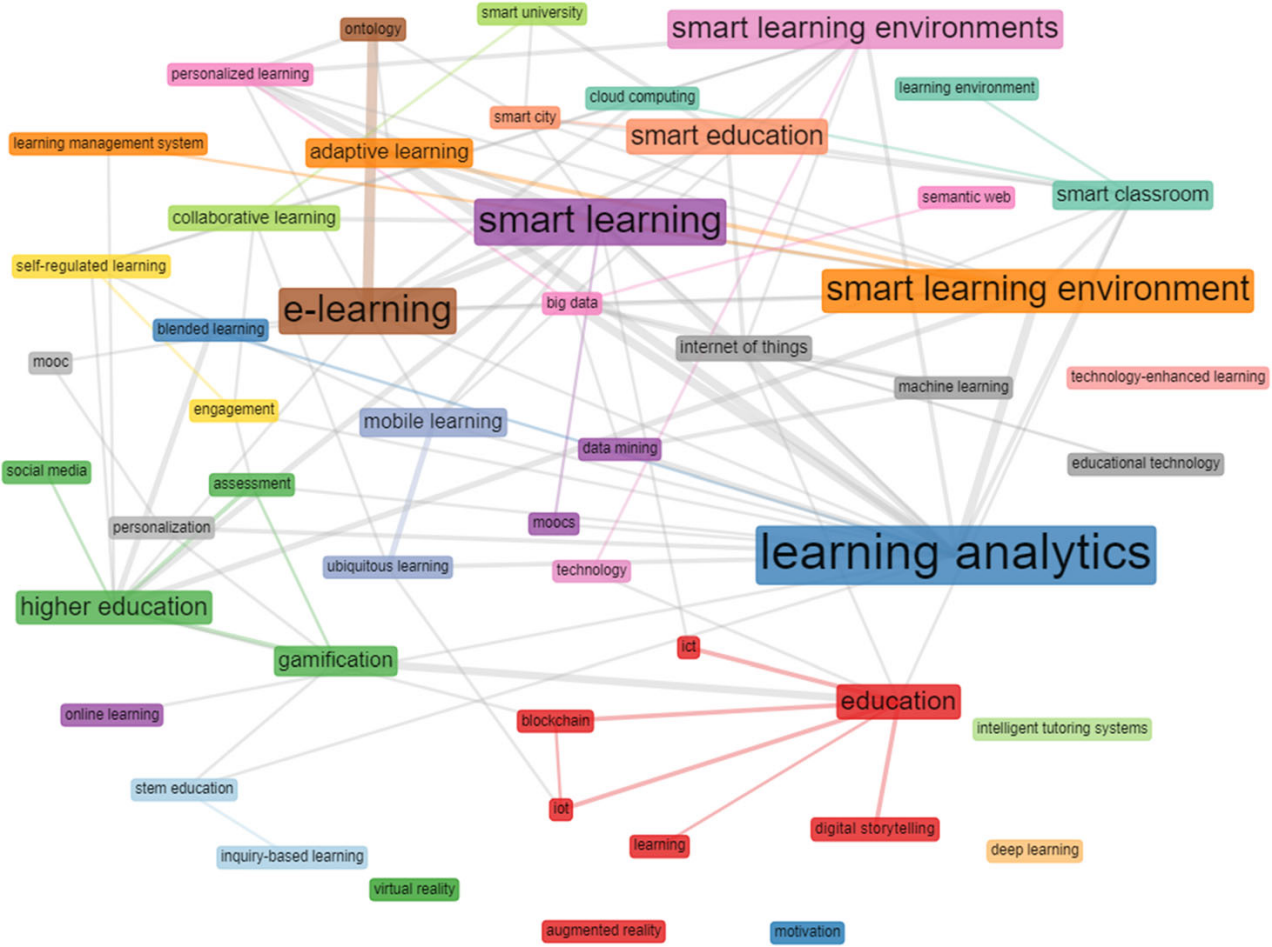
Co-occurrence network of keywords: the thicker line indicates a strong association between those keywords; thinner lines depict weak association, and keywords without connecting lines indicate that no relationship has been established

In addition, this study investigated the keywords co-occurrence network (KCN) in order to gain further insight into the trends in the field of smart learning environments. The KCN analysis presents the link between keywords in literature, which gives insight into the field’s knowledge structure (Esfahani et al., 2019). Therefore, our result shows that beyond identifying frequent keywords, as shown in the word-cloud (Fig. 8), KCN revealed the connections between them (see Fig. 10). Notably, some keywords seem to have a greater impact on a network. For example, a close examination of these keywords from its color code suggests that a bigger keyword represented by their width are cohesively connected to other smaller keywords. For instance, Education connects to digital storytelling, blockchain, IoT, ICT, and learning. Similarly, a keyword smart learning environment is closely connected to adaptive learning and learning management system.

#### Trending topics and thematic analysis of the field of smart learning environment

Furthermore, an analysis of the trending topic based on the author’s keywords from the dataset was conducted. While conducting the analysis, the following parameters were configured; timespan was set at 2011 to 2020, word minimum frequency was set to 5, number of words per year was set to 5, and word label size was also set to 5.

**Fig. 11:**
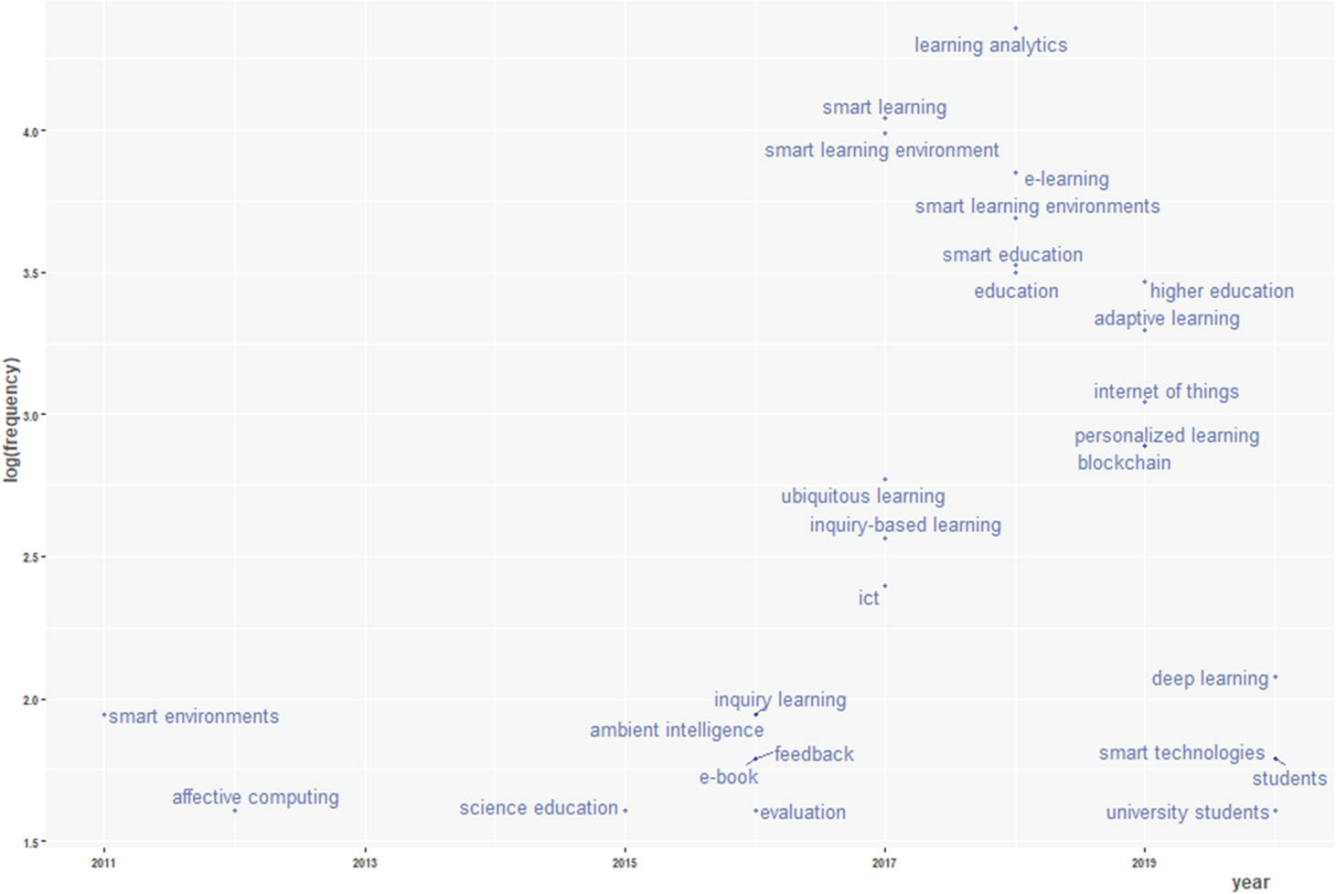
Trending topics between 2011 to 2020

Article keywords, which authors define, are usually connected to such publication content and are sufficient to derive topical aspects of a field (Song et al., 2019). This analysis gives further insight into the trending topics in terms of keywords occurrences in smart learning literature over the years. Although many authors’ keywords are shown in the word-cloud (Fig. 8), the analysis in Fig. 11 presents the hierarchical arrangement of topics in smart learning environments discussed by scholars per year. These topics could relate to the field of smart learning environments in many ways. For instance, in 2016, *inquiry learning* was the most discussed topic, and it is a pedagogical domain of smart learning environments. Similarly, in 2017, *smart learning* was the leading topic, which is a key concept of smart learning environment; in 2018, *learning analytics* was top on the list, which also formed another critical domain of smart learning environment. The result also shows that as at the time of conducting this analysis, *deep learning* remains the trending topic in 2020.

Another analysis conducted in this study is the thematic map of smart learning environments. The aim of conducting a thematic map is to gain insight into the field’s current status and what its future sustainability holds. This analysis is useful in providing knowledge to researchers and stakeholders regarding the potentials of future research development of thematic areas within a field.

**Fig. 12:**
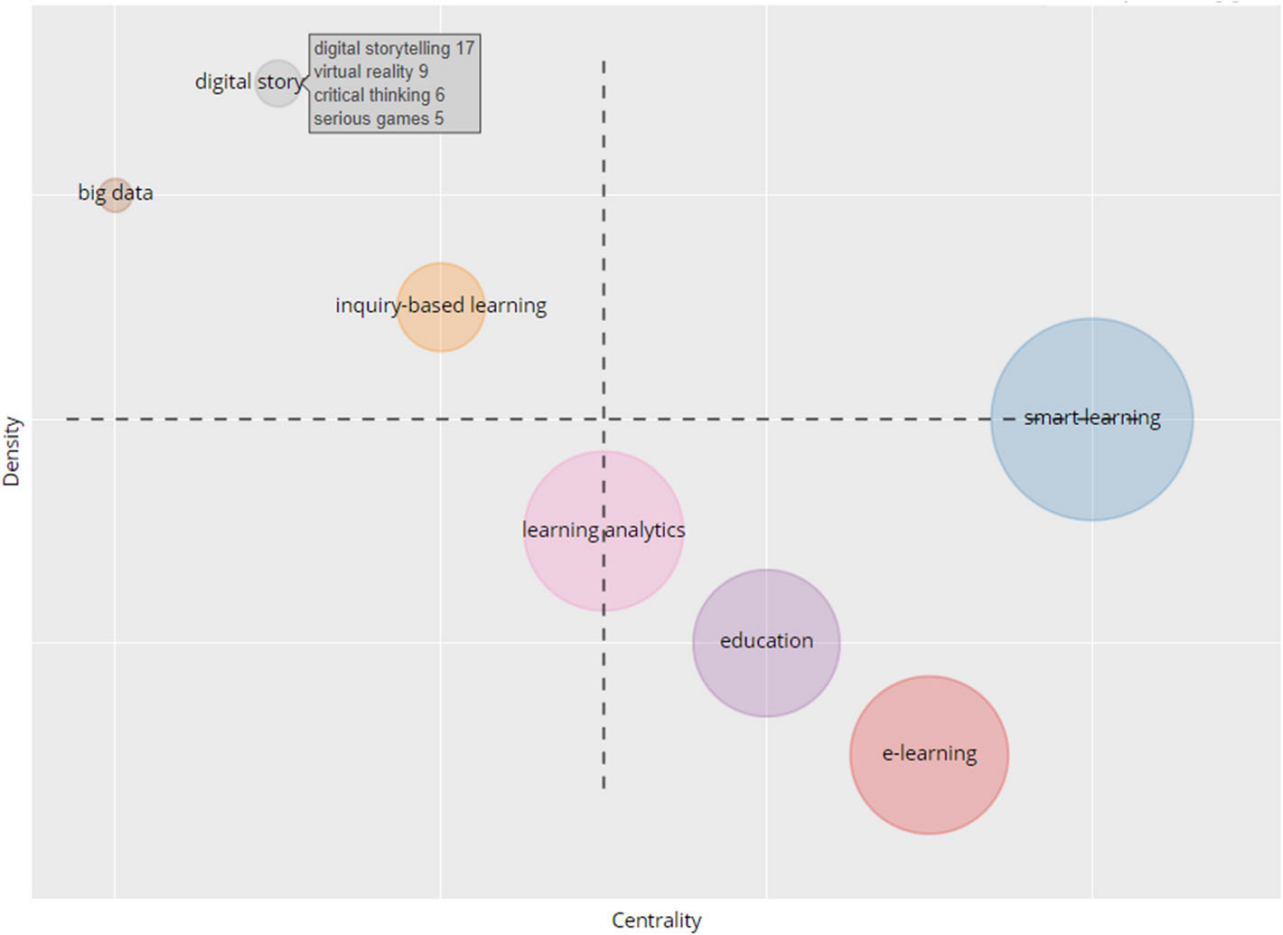
Thematic map: Q1 contains the main theme, Q2 contains highly developed and specialized themes building ties with the leading theme; Q3 contains disappearing or emerging themes; Q4 consists of foundational and transversal themes

The thematic analysis takes clusters of authors’ keywords and their interconnections to obtain themes. These themes are characterized by properties (density and centrality). The density is represented in the vertical axis, while centrality takes the horizontal axis. Centrality is the degree of correlation among different topics; density measures the cohesiveness among the nodes (Esfahani et al., 2019). These two properties measure whether certain topics are well developed or not, important or not. The higher the number of relations a node has with others in the thematic network, the higher the centrality and importance, and it lies within the essential position in the network. Similarly, cohesiveness among a node, which represents the density of a research field delineates its capability to develop and sustain itself. In Fig. 12, we provide the thematic map of the field of a smart learning environment, which is basically divided into four quadrants (Q1 to Q4).

The upper right quadrant (Q1) represents driving themes, the lower right quadrant (Q4) is underlying themes, the upper left quadrant (Q2) is the very specialized themes, and the lower left quadrant (Q3) is emerging or disappearing themes. Notably from the figure, a theme such as “smart learning,” sandwiched between Q1 and Q4, is well developed and capable of structuring the research field. In other words, smart learning remains the leading theme within the field. Themes such as “education” and “e-learning” seen in Q4 are the basics and are very important for the field’s development. Themes in Q2 have developed internal bonds but still of marginal contribution to the development of the field of smart learning. This finding suggests that themes in Q2 such as storytelling, virtual reality, critical thinking, and serious games, are potential topics that need to be more connected to smart learning. Scholars in this field may explore these pedagogical tools (storytelling and serious games) and technological approach (virtual reality) to provide smart learning environments for a twenty-first-century learning experience.

The theme in Q3, “learning analytic,” appears to be emerging but transverses Q4, indicating that some of its components are basic and necessary for developing the field of smart learning environments. The thematic analysis suggests that more efforts are needed to develop themes such as “digital storytelling” and its associated components such as virtual reality, critical thinking, and serious games, to establish more ties with “smart learning”. This effort is necessary because digital storytelling, an established field, can significantly contribute to the smart learning environment’s structure, future, and sustainable development.

## Conclusions

This study has tried to provide an extensive review of scientific publications in the field of smart learning environment over time using bibliometric analysis. The study investigated the themes of smart learning in the publications; recognized prolific scholars and their contributions; explored social networks and collaborations across institutions, countries, and regions over time, and presented the thematic analysis of the field of smart learning environments by showing its current status regarding the themes, and future prospects. A total of 1081 documents were retrieved from the Scopus database for this study. This work makes a number of prominent contributions to the research body. First, the study revealed that the first paper on smart learning environments was published in 2002, which perhaps signifies the beginning of the field of the smart learning environment. Relevant publishing outlets were identified in this study. Foremost among the publishing sources as revealed by the study is the “Computers & Education.” This result provides an important guide to scholars regarding the publishing outlet that is suitable for their research papers.

Additionally, an investigation into relevant articles published in the field revealed that the work of Kinshuk et al. (2016) stands out; these authors work mainly focused on the transformation of technology-enhanced learning into smart learning environments. Perhaps, their work sets the stage for discussions on the features and characteristics of smart learning environments from the technology and pedagogy perspectives. Similarly, our result delineates that the United States has the highest number of scientific productions in the field of smart learning environments over the years. That suggests that the United States remained the most relevant country in the field of smart learning environments. Regarding institutions’ contributions and relevance, Beijing Normal University in China tops the list. In the aspect of prolific scholars making an immense contribution to the field of smart learning environments, Arthur C. Graesser from the United States tops the list with an h-index of 8. Besides, scholars such as Kinshuk, Graesser, Ogata, De Jong, and Aguilar have established a wide range of collaboration networks.

Furthermore, the study revealed that the field of smart learning environments is recently evolving with the emerging and growing aspects such as “learning analytics,” “adaptive learning,” “personalized learning,” “blockchain,” and “deep learning”. The thematic analysis results show that themes such as “digital storytelling” are emerging and connected to smart learning environments. However, this theme and its associated components, such as virtual reality, critical thinking, and serious games, needs to be further developed to establish more ties with “smart learning”. The study further showed that in the mid-year of 2020, “deep learning” remains the trending topic. It is interesting to discover that between 2017 and 2020, newer topics connected to artificial intelligence (AI) such as learning analytics, blockchain, and deep learning, have emerged and grown to become research hotspots in smart learning environments. These findings underscore the importance of deepening further studies to leverage AI in future designs of smart learning environments. As part of our conclusion, some suggestions for future research in the field of smart learning environments are highlighted in this study.

1. It could be essential to develop more extensive research collaborations between scholars and institutions, thereby creating a more global impact on smart learning environments’ potentials for an enhanced learning experience.
2. It is suggested that scholars invest more effort in learning analytics, machine learning, and deep learning, as the study shows that they are future research topics in smart learning environments.
3. More effort into researching digital storytelling, serious games, virtual reality, and critical thinking by educational technologists and designers of smart learning environments is suggested. This study has shown that there are potentials to adopt these strategies in developing twenty-first-century learning.

### Study limitations

This study has some limitations. Majorly, the study weakness is about the sample data collection. The study encountered a technical limitation in terms of the software used to conduct the analysis, where the merging of data from different databases was not possible at the time the study was conducted. The sample in this study was collected from the Scopus database, which may result in missing out relevant data. Collecting sample data from multiple independent databases would certainly improve the study in a significant way. In addition, the search keywords used in querying the database could be improved to consist more relevant keywords. This limitation should motivate future work where scholars could explore ways of collecting data from multiple databases with expanded keywords for a more in-depth analysis.

In sum, we conclude that this study hopes its findings will provide insight to researchers, specifically, the young scholars in smart learning environments regarding the research landscape and future research hotspots. For example, young researchers who are beginning to research in the field can quickly identify top articles, prolific authors, and research hotspots in the field of smart learning environments. In addition, the study shows emerging topics in the field of smart learning environments, which needs to be further developed to connect to the objective of smart learning. Findings from this study provide a quick overview of the output in this field over the years and relevant pointer to the future direction in the field of smart learning environments.

## Data Availability

The datasets generated during this study are available from the corresponding author.

## Abbreviations

SLE: Smart Learning Environments
RQ: Research Questions
PY: Published Year
TC: Total Citation
KCN: Keywords Co-occurrence Network

## Footnotes

1 https://bibliometrix.org/
